# Digital Technology Use and Dementia Risk in Older Adults: Evidence from NHATS

**DOI:** 10.1093/geroni/igaf122.516

**Published:** 2025-12-31

**Authors:** Wenxuan Huang, Jiao Yu, Roland Thorpe

**Affiliations:** Johns Hopkins University, Baltimore, Maryland, United States; Yale University, New Haven, Connecticut, United States; Johns Hopkins Bloomberg School of Public Health, Baltimore, Maryland, United States

## Abstract

Digital technology has become integral to older adults’ lives, supporting healthcare, social, and leisure needs. While the cognitive benefits of technology use have been documented in recent research, existing studies are limited by cross-sectional designs, narrow measures of technology engagement (e.g., single domains like computer use), and small sample sizes. Furthermore, no prior research has explored how this relationship varies between older adults with and without activity limitations. Leveraging 12 years of longitudinal data from the National Health and Aging Trends Study (NHATS; n = 7,027), this study investigates associations between three domains of technology use—general device use (e.g., smartphones), everyday technology use (e.g., emailing, online shopping), and digital health technology use (e.g., telehealth)—and dementia incidence among community-dwelling older adults. Analyses adjust for selection bias using inverse probability weighting and stratify by three activity limitation categories (mobility limitations, self-care limitations, household limitations). Weighted mixed effects logit model results indicate that everyday technology use related to routine activities (i.e., emailing/texting, online shopping) is associated with an approximately 80% lower odds of dementia, compared to 48% and 64% respectively for using any technology devices and any digital health technology. Stratified model results demonstrate significantly greater marginal effects for older adults with activity limitations, suggesting that they may glean more cognitive health benefits from technology use than their limitation-free counterparts. These findings underscore digital technology’s potential as a scalable, modifiable intervention to reduce dementia risk, particularly for vulnerable populations with disabilities.

